# Genetically modified microorganisms for agricultural use: an opportunity for the advancement of risk assessment criteria in Argentina

**DOI:** 10.3389/fbioe.2025.1612226

**Published:** 2025-06-13

**Authors:** Clara Rubinstein, Gabriela Levitus, Carmen Vicien, Natalia Andrea Modena, Sandra Ruzal, Facundo Vesprini, Dalia Marcela Lewi, Cecilia Caminoa, Maria Fabiana Malacarne, Nerina Francescutti, Juan Ignacio Amaturo

**Affiliations:** ^1^ Institute for Scientific Cooperation on Health and the Environment (ICCAS), Buenos Aires, Argentina; ^2^ Argentine Association for the Development and Education in Biotechnology (ArgenBio), Buenos Aires, Argentina; ^3^ School of Agriculture, University of Buenos Aires, Buenos Aires, Argentina; ^4^ Bayer Crop Science, Buenos Aires, Argentina; ^5^ Biochemistry Institute, Exact and Sciences School, University of Buenos Aires, Buenos Aires, Argentina; ^6^ Instituto Nacional de Tecnología Agropecuaria, Buenos Aires, Argentina; ^7^ BASF SA, Buenos Aires, Argentina; ^8^ Argentine Seed Association, Buenos Aires, Argentina; ^9^ Corteva Agriscience SRL, Buenos Aires, Argentina

**Keywords:** microorganisms, bio-inputs, biologicals, risk assessment, biosafety

## Abstract

The development and use of biologicals in agriculture is of growing interest globally. The potential of these tools to increase and protect yield complementing other tools has stimulated the interest of developers. Agricultural countries like Brazil and Argentina in Latin America have extensive experience with the use of biologicals for biocontrol and as seed inoculants. The last decade has seen the number of bio-based startups grow in the region, many of those dedicated to the development of microbial based bio-inputs. The potential for improving the efficacy and functionality of these products by means of gene technologies is very promising; however, the regulatory oversight of these innovations needs adaptation to become fit for purpose. The Biotechnology Working Group at ICCAS identified the need for a science-based discussion on this matter and considered alternatives to the current paradigm, developed over 30 years ago for transgenic plants.

## Introduction

The Institute for Scientific Cooperation on Health and the Environment^1^ (ICCAS in Spanish) is a scientific non-for-profit association based in Argentina, which brings together experts from academia, industry and government and provides a neutral forum to discuss scientific matters of public interest. The Biotechnology Working Group has over 25 years of existence driving numerous capacity building programs in the Latin American region, hosting scientific discussions on biosafety criteria and developing conceptual tools for the risk evaluation of products derived from modern biotechnology (Garcia-Alonso et al., 2014; Beker et al., 2016; Fernandez Ríos et al., 2018; Capalbo et al., 2020; Vesprini et al., 2020). This working group identified a need for a scientific discussion to explore science-based approaches adapted to the case of genetically modified microorganisms (GMM) for agricultural use, with focus on bacteria.

Initially, discussions touched on the appropriate safety measures to conduct experimental field trials with GMM under the current biosafety paradigm applied in Argentina, originally developed for GM plants. However, the profoundly different nature of microorganisms as compared with plants - microorganisms are not sessile and the genetic exchange mechanisms between them are completely different and diverse - led to a more general question about how risk assessment criteria should be applied to these cases, even beyond experimental releases. It was clear then, that in order to facilitate the safe deployment of these innovations, an adaptation of the assessment criteria was required to become fit for the purpose of the microbial world. Similar discussions are also taking place in other regions and venues (OECD, 2024b), as biological tools are increasingly becoming part of sustainable agriculture strategies, and the challenges to use the safety assessment criteria created for plants become evident.

The present work reflects the result of these discussions, intends to contribute to a science-based approach adapted to the nature of these products - which are not chemicals nor plants - and bring to light the need for a paradigm shift to assess their biosafety. The aim of this work is to present specific aspects of the biology and genetics of microorganisms (in particular bacteria) that are relevant to risk assessment and management.

## Use of microbial based bio-inputs in the region

Different functional groups of microorganisms are applied to agricultural production. Biofertilizers promote growth in plants through nitrogen fixation or phosphorus solubilization mechanisms. Nitrogen-fixing bacteria can be free-living, endophytic, or nodulating (Laranjo et al., 2014; Silva et al., 2023; Kramer et al., 2020). Phytostimulants include phytohormones producers or promoters of plant growth through direct mechanisms. Biocontrol agents, on the other hand, include the so-called microbial biopesticides, which act through the production of larvicidal toxins, bacteriocins, biosurfactants, antibiotics or cell wall-degrading enzymes. Other biocontrol mechanisms involve inhibition of the quorum sensing of the pathogens or the induction of systemic resistance in the host plants (Gómez-Godínez et al., 2023; Legein et al., 2020).

Nitrogen -fixing inoculants make up around half of the global biofertilizer market, while over 55% of the globally marketed biopesticides are microbial (Aramendis et al., 2023); Europe and Latin America are the top users. Over 20 million hectares of soybean are planted every year in Argentina, most of which are treated with over 25 million doses of biofertilizers. In 2022, close to 25 million doses of formulations based on *Azospirillum* sp. were used in Argentina and Brazil for the treatment of corn, soybean, peanuts, common bean, wheat, sorghum, sunflower and horticultural production (Barbosa et al., 2021; Compant et al., 2025).

Argentina has a 40-year history of research, development and agricultural use of bio-inputs and it was one of the first countries to release a commercial product containing an *Azospirillum brasilense* strain back in 1996 (Cassan and Diaz Zorita, 2016). Biocontrol agents have been used in Latin America since the late 19th century and are currently used on a large scale, being the region with the largest historical adoption (Biaggioni et al., 2013; Gottems, 2021).

Latin America is also a hub for innovative startups with Argentina, Brazil, and Chile leading in the biotechnology sector thanks to the large research community and the relevance of agricultural and food chain applications in these countries (Peña and Jenik, 2023).

Microbial formulations are subject to biotic and abiotic factors that affect their performance, stability or consistency in the fields. Both classical and emerging strategies are applied by developers and researchers to address these problems (Batista and Singh, 2021), with gene technologies having great potential, although these will require adaptive risk assessment criteria.

## Regulatory context for conventional bio-inputs in Argentina

The National Service for Agri-food Health and Quality (SENASA) is the responsible agency for the registration of these products and has recently issued an updated normative for biological pesticides and fertilizers (SENASA, 2023). Within this framework, experimental releases of conventional microbial bio-inputs are not subject to a regulatory permitting process for proof of concept, selection of candidates or efficacy testing purposes, among others. However, for imported microorganisms, authorizations to introduce samples for testing are needed and the amount requested for each trial needs to be specified. A characterization of the imported microorganism is also required, focused on pathogenicity, toxicology and eco-toxicology. The most frequently requested microorganisms for import are viruses for biocontrol and plant growth promoting bacteria. In all cases, for commercial registration, a complete data package for safety assessment is required.

## The case of genetically modified microorganisms (GMM)

Argentina has extensive experience with the risk assessment of biotechnology derived products. CONABIA (the National Advisory Committee for Agricultural Biotechnology) was created in 1991 and was the first of its kind in the Latin American region. Ministerial Resolution 763/2011^2^ rules the oversight of GMO and both CONABIA and the Biotechnology Food and Feed Safety Coordination at SENASA are involved in the regulatory assessment process for commercial authorization (CIB GM crops, 2025).

An environmental risk assessment for GMO is currently implemented, originally developed for GM plants and updated over time. The risk assessments for both contained/confined activities and commercial production are carried out by the technical staff of the Biotechnology and Innovation Coordination at the Agriculture, Livestock and Fisheries Secretariat and by CONABIA^3^.

A specific guideline for GMM has been developed following the same model (Resolution 5/2018 and Resolution 52/2019) and is presently being updated to consider the scientific and technological state of the art (CIB GMMs, 2025). So far, several GMM for industrial uses or vaccines have been approved, but no GM bio-inputs have been authorized for experimental or commercial environmental release in Argentina^4^.

## Genetically modified microorganisms (GMM) and scientific risk assessment criteria

The WG discussions focused on the unique challenges posed by GMM, as the profound differences between microorganisms and higher organisms makes it very difficult to apply the same criteria developed over 30 years ago.

Microbial diversity is a dynamic phenomenon resulting from highly plastic adaptation processes mediated by mutations, exchanges and horizontal gene transfer events. Genetic exchange mechanisms have been well characterized in microorganisms, with conjugation, transduction and transformation being the main ones (Arnold et al., 2022; Magnabosco et al., 2024). Conjugative transfers of broad-host range plasmids and transformation of chromosomal genes occur in different environments, including in planta, also between remotely related microorganisms (Kay et al., 2003). For these reasons, defining species is not trivial for microorganisms and thus it is not appropriate to apply the same logic to interpret phylogenetic relations used for higher organisms (Kunin et al., 2005; OECD, 2003), or to refer to the “compatible species” paradigm to assess potential risks of gene dispersion.

This said, it is important to also consider that there are natural barriers to exogenous DNA (restriction-modification, CRISPR-like and related mechanisms) and that GMM are generally unfit to survive and multiply in nature due to several factors (expression burden, genomic disruption, domestication). Mutations, chromosomal rearrangements and other mechanisms can improve microorganisms for bioproduction purposes but make them generally less fit in the environment, where local microbiota can act as an ecological barrier (Steensels et al., 2019).

The environmental release of GMM is not new. Since the first field trials to evaluate GM *Pseudomonas syringae* (“Ice minus” strain) in the 1980s, GMM have been investigated for decades by both academia and industry (Ke et al., 2021; De Leij et al., 1995; Wilson and Lindow, 1994). Laboratory and field research in experimental plots made it possible to monitor and trace modified bacteria to assess their survival, dispersion and effects on the local microflora. This research showed no relevant differences between the modified bacteria and their parental strains in terms of survival, spread or persistence, and observed effects on resident microflora were transient, or limited and less pronounced than those induced by conventional agronomic practices (Amarger, 2002; Chemla et al., 2025).

## Back to the basics: comparative approach, the familiarity concept and the issue with the definitions

 As Dr Hiroshi Yoshikura stated in the context of the OECD workshop held in 2015^5^ to discuss this topic: *“One approach could be going back to the two complementary concepts developed by OECD in early 1990s: familiarity and substantial equivalence”* (Yoshikura, 2015). This recommendation seems the most reasonable to enable an evidence-based risk assessment of GMM, as the conceptual framework based on the fundamental pillars developed for Modern Biotechnology can be adapted to the particular biology of microorganisms without compromising the robustness of the biosafety assessment (OECD, 2015). The comparative approach, developed decades ago for biotechnology derived plants (OECD, 1986), was considered and still is the most robust approach to establish the “substantial equivalence” of the new organism compared with a “*conventional counterpart with a history of safe use*” (OECD, 1993b; Codex, 2003), also considering familiarity as proposed back in 1993 as an essential part of the Problem Formulation process (OECD, 1993a; Capalbo et al., 2020).

Another key element to consider is the regulatory definition of a GMM. GMO definitions are not harmonized and different versions or interpretations bring additional complexities (De Schrijver et al., 2024). In fact, depending on the definition, microorganisms improved by classical genetics or techniques resulting in changes that could have been obtained through classical genetics, could end up being categorized as GMM. Gene technologies that may introduce specific regulatory sequences or leave non coding structural traces (“scars”) in the genome could end up being subject to the regulatory oversight for GMO if unfit definitions are in place (Chemla et al., 2025).

Historically, it was generally accepted what a GM plant or animal was, until the advent of gene editing disrupted this virtual consensus (Podevin et al., 2012) and this is now further disrupted with the need to re-think what a GM microorganism is. As Lensch et al. (2024) have recently pointed out, “*The terms GMOs and non-GMOs are no longer fit for purpose. Even more clearly than in plants, the boundaries between “genetically modified” and “conventional” microorganisms have become blurred*”.

The assessment approach discussed in the next section intends to apply the logical processes of Problem Formulation and the Paths to Harm to the risk assessment of GMM, focusing on the equivalence of the GMM with the host microorganisms and considering the degree of familiarity with the hosts, the environment, the trait and the intended uses.

### Proposal for the identification of acceptable risks for the environmental release of GMM: a fit for purpose approach

The WG addressed some questions about the evidence needed to evaluate the risks for the environmental release of GMM. Some key considerations were firstly identified to frame the discussion, namely:

• Given the available knowledge of microbial genetics, physiology and metabolism, and the analytical methodologies used in microbiological research (bioinformatics, high throughput DNA sequencing and metagenomics, cultivation-independent community analyses, novel cultivation methods, antimicrobial sensitivity assays, among others), in most cases a complete characterization can be generated under laboratory and/or greenhouse conditions for conventional or GM microorganisms (Janssen et al., 2002; Stevenson et al., 2004)
• Current registration requirements for conventional agricultural bio-inputs cover the majority of relevant biosafety aspects, like toxicity, pathogenicity, antibiotic resistance or production, among others; so, these are not unique to GMM.
• Generally, the main objective of experimental field releases of bio-inputs is to test efficacy.

With these in mind, three main questions were discussed:

- *What information is essential to make a decision about the risk of releasing a GMM?*
    The required information should derive from a sound Problem Formulation and the Paths to Harm exercises (see below).
- *Which biosafety related endpoints would be measured in the field that could not be measured in lab or greenhouse studies?*
    Even when a complete characterization of the host microorganisms as well as the trait incorporated in the GMM can be achieved during the discovery/development/design phases, on a case by case basis and hypotheses driven, some endpoints could require field trials to be measured.
- *When needed, which management measures should be applied to experimental releases of GMM?*
    Measures like limited acreage, buffer zones, distances from commercial crops, etc., can be implemented based on the risk hypotheses identified. The life cycle, the mode of action and the intended use (i.e., vegetative vs. sporulating microorganisms, free living vs. symbiont or endosymbiont, inoculation vs. foliar spraying, etc.) will determine if additional measures such as drift reduction or monitoring might be required, based on plausible risk hypotheses.

## Problem formulation and the paths to harm for genetically modified microorganisms (GMM)

The Problem Formulation (PF) process is a well-established methodology for risk assessment currently applied by numerous agencies and risk assessors globally (Garcia-Alonso, 2013).

The process starts by framing the case and defining the scope of the assessment focused on the protection goals that are relevant to the case. The second step involves gathering the available knowledge on the case under review and related cases. In fact, considering what is known (familiar) is extremely important. In the case of GMM, a thorough characterization along with the degree of familiarity with the host microorganisms and the novelty of the expressed traits will guide the assessment.

The central step in PF is the identification of plausible and testable risk hypotheses (defined as scientific hypotheses specifically focused on the risks of adverse effects to the relevant protection goals), based on the information that is already available and the similarity to the non-modified host (comparative approach).

Going back to protection goals, these are not always defined in policies, however, there are general goals that can be a starting point and provide the basis for the process. This said, more refined, operative goals are needed to be able to assess relevant exposure scenarios (Garcia-Alonso and Raybould, 2014).

Some globally established protection goals are human and animal health, agricultural production and ecosystem services. Operative goals are generally related to the protection of beneficial organisms and the preservation of commercially important crops. Having clear protection objectives is key to performing robust, evidence-based risk assessments.

Once relevant operative goals are defined and risk hypotheses identified, the last step is to analyze the possible paths to harm under probable exposure scenarios in order to verify which hypotheses are of possible occurrence and establish a study plan to test them if there is not available data (OECD 2024b; Gray, 2012).

**FIGURE 1:**
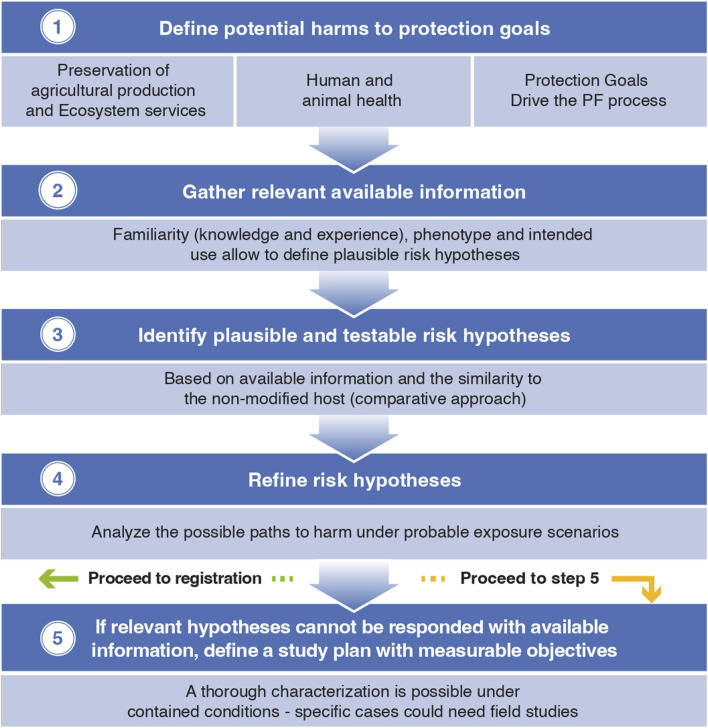
Problem Formulation Sequence applicable to GMM.

In summary, applying the comparative approach based on PF would provide an adequate evidence base for decision-making and enable the safe deployment of bio-inputs based on GMM. In cases where this process identifies the need for field trials, the data packages generated in lab-greenhouse studies and the familiarity with the host and traits should allow safe experimental releases with reasonable management measures (see Figure 1)

Considering all the above, the general conclusion was that releases for experimental purposes (like efficacy testing) can be allowed with an appropriate risk assessment based on available information. As other trials subject to regulatory oversight, these would require to provide a detailed protocol with evaluation objectives, management of the trial from planting to harvest, etc.

Upon reporting of the trials results and provided no additional concerns are raised, a final decision on the biosafety of the GMM should be possible. With this, the registration process would proceed as with conventional bio-inputs, which -as above detailed-need to provide a complete safety data package.

## Discussion: a paradigm shift is in order

Following a sound PF process for the defined protection goals is the best science-based way to identify potential risks and any missing data that might be needed to release a GMM. In contrast to plants, where field experiments are needed for event selection and characterization, and for which confinement conditions are well defined and managed, a complete dataset for the characterization of microorganisms can be generated in laboratory and greenhouse studies.

If the necessary information identified during PF is available, experimental releases would be possible with basic management measures, as discussed. Once established that the GMM will not introduce new risks to health or the environment, the safety assessment of the bio-inputs based on the GMM could follow that of conventional strains for registration.

As noted, the advent of gene editing tools revealed the need to revisit the risk assessment criteria and the definition of a GMO, and GMM are now renewing this challenge. The GMM status depends on the definitions in use and the current one has been developed with higher organisms in mind. A new definition would need to be developed for microorganisms based on criteria that consider the particular nature of their biology.

When considering biosafety, design strategies are and will be critical to ensure safe releases of GMM. Biocontainment strategies currently available and under development can provide higher levels of biosafety when appropriate: besides genomic insertions-considered good general practice-auxotrophy, transcriptional control, gene entanglement and xenobiology are some examples (Gómez-Tatay and Hernández-Andreu, 2024; Chlebek et al., 2023; Chemla et al., 2025). Also, noteworthy, advanced technologies are transforming the research and development of biologics, combining big data and artificial intelligence to design innovative products, which will require adaptive, fit for purpose criteria (Wang, 2025).

Changing the current paradigm would be an important contribution to the development of innovative bio-inputs, which can be delayed if field testing with GMM is perceived as not possible due to the difficulties to meet requirements, in particular by small companies, public sector or startups (Chemla et al., 2025; Thakor and Charles, 2025). Finally, interdisciplinary work and regulatory pre-consultations, as is implemented in Argentina, are extremely important for both developers and regulators, as these instances inform regulators, allow for guidance to developers and add transparency to the whole process (OECD, 2022; OCED 2024a).

## Data Availability

The raw data supporting the conclusions of this article will be made available by the authors, without undue reservation.

## Footnotes

1
https://iccas.org.ar/en/home

2
https://servicios.infoleg.gob.ar/infolegInternet/anexos/185000-189999/185806/norma.htm

3
https://www.argentina.gob.ar/agricultura/bioeconomia/biotecnologia/documentos-de-decision-conabia

4
https://www.argentina.gob.ar/microorganismos-gm-con-autorizacion-comercial

5 Hiroshi Yoshikura, National Institute of Infectious Diseases, Ministry of Health, Labour and Welfare, Japan.
